# Transforming Cardiac Imaging: Can CT Angiography Replace Interventional Angiography in Tetralogy of Fallot?

**DOI:** 10.3390/jcm14051493

**Published:** 2025-02-23

**Authors:** Ali Nazım Güzelbağ, Serap Baş, Muhammet Hamza Halil Toprak, Demet Kangel, Şenay Çoban, Selin Sağlam, Erkut Öztürk

**Affiliations:** 1 Department of Pediatric Cardiology, Saglik Bilimleri University, Basaksehir Cam and Sakura City Hospital, Istanbul 34480, Turkey; muhammedhamzatoprak@hotmail.com (M.H.H.T.); demetdasdemir90@gmail.com (D.K.); snycbn84@gmail.com (Ş.Ç.); erkut_ozturk@yahoo.com (E.Ö.); 2 Department of Radiology, Saglik Bilimleri University, Basaksehir Cam and Sakura City Hospital, Istanbul 34480, Turkey; serapbas579@gmail.com; 3 Department of Anesthesiology, Saglik Bilimleri University, Basaksehir Cam and Sakura City Hospital, Istanbul 34480, Turkey; selinsaglams@gmail.com

**Keywords:** tetralogy of fallot, computed tomography angiography, transthoracic echocardiography, congenital heart disease, preoperative imaging

## Abstract

**Background:** Tetralogy of Fallot (TOF) is a complex congenital heart condition characterized by four major anatomical abnormalities. Accurate preoperative imaging is critical for optimal surgical outcomes, with transthoracic echocardiography (TTE), computed tomography angiography (CTA), and conventional catheter angiography (CCA) being the primary diagnostic tools. This study aimed to compare the diagnostic utility of TTE, CTA, and CCA in preoperative evaluations of TOF patients, focusing on anatomical parameters, imaging accuracy, and patient outcomes. **Methods:** A retrospective, single-center analysis included TOF patients under one year of age who underwent complete repair between January 2021 and December 2024. Preoperative imaging with TTE, CTA, and CCA was analyzed for parameters including pulmonary artery diameters, Nakata index, McGoon ratio, and Z-scores. Radiation exposure, procedure duration, contrast volume, and complications were documented. Statistical analyses were performed to assess the comparative accuracy and safety of these modalities. **Results:** All patients underwent TTE (n = 127), while CTA was performed in 86 patients and CCA in 41 patients. Among 127 patients, 62% were male, with a mean age of 5.81 ± 2.15 months. On TTE, CTA and CCA provided statistically similar measurements of the pulmonary annulus, main pulmonary artery, and branch diameters, with no significant differences in the Nakata index and McGoon ratio. CTA had a shorter procedure duration (3.1 ± 0.58 min) and lower radiation dose (1.19 ± 0.22 mSv) compared to CCA (20.73 ± 11.12 min; 5.48 ± 1.62 mSv). CTA successfully identified major aortopulmonary collateral arteries (MAPCAs) in 10% of patients and detected additional pulmonary pathologies, such as subsegmental atelectasis in 12%. Access site complications were observed in 10% of CCA cases but were absent in CTA evaluations. **Conclusions:** CTA emerges as a highly effective and non-invasive alternative to CCA for preoperative assessment of TOF, offering comparable anatomical accuracy with significantly reduced procedural risks, radiation exposure, and contrast volume. Combining TTE and CTA provides comprehensive diagnostic coverage, minimizing the need for invasive procedures and enhancing surgical planning. These findings underscore the evolving role of CTA in the management of congenital heart disease, contributing to improved patient safety and outcomes.

## 1. Introduction

Tetralogy of Fallot (TOF) is one of the most complex congenital heart diseases, and it is the most common cyanotic heart condition observed in infants [1]. TOF is characterized by four primary anatomical abnormalities: ventricular septal defect (VSD), right ventricular outflow tract (RVOT) obstruction, an overriding aorta, and right ventricular hypertrophy. TOF manifests through severe cyanotic episodes in infancy. Given the diverse anatomical manifestations of TOF, each patient requires a detailed anatomical assessment, which necessitates the use of advanced imaging techniques [2].

The timing of TOF repair requires a multidisciplinary approach, balancing the risks of early versus delayed surgery while optimizing outcomes tailored to each patient’s clinical and anatomical characteristics. Advances in surgical techniques and perioperative care continue to improve outcomes, making early repair feasible and beneficial for the majority of patients with TOF. The American Heart Association (AHA) and the European Society of Cardiology (ESC) recommend complete repair of TOF within the first year of life, ideally between 3 and 6 months of age, for most patients. Neonatal repair may be performed in cases of severe cyanosis or life-threatening hypoxic spells, while staged procedures may be considered in select cases with complex anatomy [3]. Historically, the diagnostic and preoperative assessment of TOF heavily relied on invasive cardiac catheterization and angiography. These techniques were instrumental in providing detailed evaluations of vascular structures, including the pulmonary arteries, coronary arteries, and other major cardiovascular structures. However, due to the invasive nature of cardiac catheterization, it carries certain risks and may place considerable stress on patients. As a result, there has been an increasing demand for less invasive diagnostic techniques that can provide similarly detailed anatomical insights without the associated risks of invasive procedures. Complications such as infection, bleeding, arterial injury, and even mortality, although rare, underscore the need for alternative, safer methods [4].

In recent years, computed tomography angiography (CTA) has emerged as a superior and non-invasive alternative to traditional invasive angiography [5]. CTA offers significant advantages, particularly for pediatric and high-risk patients, due to its non-invasive nature, faster acquisition times, and high-resolution imaging capabilities. With the development of multidetector CT scanners and advanced post-processing techniques, CTA now allows for rapid and detailed evaluation of both cardiovascular and non-cardiovascular structures, which plays a crucial role in surgical planning. Compared to invasive angiography, CTA provides superior imaging of the pulmonary arteries, coronary arteries, and smaller pulmonary vessels while also minimizing the risks and discomfort associated with traditional catheterization [6].

Naveen Garg et al. demonstrated that multidetector computed tomographic (MDCT) angiography is a reliable, non-invasive alternative to cardiac catheterization angiography for preoperative evaluation of TOF patients over five years of age [7]. Additionally, Amarendu Kumar et al. demonstrated that in unrepaired TOF patients weighing more than 10 kg, CTA-acquired pulmonary vascular dimensions provide reliable assessments. [8]. The use of CTA has become increasingly prominent in the preoperative evaluation of TOF patients. It allows for the precise identification of major aortopulmonary collateral arteries (MAPCAs), as well as the detection of extrinsic vascular tracheobronchial compression, both of which are essential for surgical planning. This high level of anatomical detail enables surgeons to approach surgery with a more comprehensive understanding of the patient’s unique anatomy. Moreover, CTA reduces the need for sedation or even eliminates it entirely, particularly in pediatric patients, further enhancing patient comfort during the imaging process [9].

In the postoperative setting, CTA is equally valuable, especially when magnetic resonance imaging (MRI) is contraindicated or suboptimal. Conditions such as the presence of metallic implants, pacemakers, or claustrophobia may prevent the use of MRI [10,11]. In such cases, CTA serves as an effective alternative, providing detailed assessments of graft materials, vascular calcifications, and other postoperative changes. The rapid imaging times offered by CTA also minimize patient discomfort during the scanning process, which is particularly beneficial for pediatric TOF patients, ensuring a smoother and less distressing experience [12].

One of the primary reasons CTA has gradually replaced invasive cardiac catheterization in TOF patients is the inherent risks and prolonged recovery associated with invasive procedures. Invasive cardiac catheterization not only carries the risk of complications but also often necessitates a longer recovery period for patients. In contrast, CTA offers faster results and causes minimal discomfort. The high-resolution images generated by modern CTA technology provide surgeons with detailed and accurate anatomical information, enabling them to better understand the complexities of the patient’s anatomy and make more informed surgical decisions. As a result, CTA has contributed to improved surgical outcomes, enhanced survival rates, and more effective long-term follow-up for TOF patients. In conclusion, the shift from invasive cardiac catheterization to CTA represents a significant advancement in the diagnosis and surgical planning of TOF patients. CTA offers a safer, faster, and more detailed method of imaging, allowing for more precise preoperative and postoperative assessments. The increasing use of CTA has revolutionized the management of TOF, leading to an improved understanding of its complex anatomy, better surgical outcomes, and overall improvements in patient quality of life [13]. In our study, we compared the preoperative imaging results of CCA, which we used initially, and CTA, which we adopted later for TOF patients, as well as their postoperative outcomes over time.

## 2. Material and Methods

Our study was a single-center, retrospective study. We included patients under one year of age who underwent complete surgical repair between January 2021 and December 2024. In our study, we included standard TOF cases that underwent early complete repair, focusing specifically on patients who underwent surgery under the age of one year. This is consistent with the American Heart Association (AHA) and European Society of Cardiology (ESC) guidelines, which recommend TOF repair within the first year of life, preferably between 3 and 6 months of age, as early surgical intervention has been shown to improve pulmonary artery development and long-term survival. To ensure a more uniform study population, we excluded certain TOF subtypes, such as TOF with pulmonary atresia, due to their different pathophysiological characteristics and management strategies. The following exclusion criteria were applied:

- Patients over one year of age
- TOF patients with pulmonary atresia
- TOF patients with atrioventricular septal defect
- TOF patients with hypoplastic ventricles
- Patients who had previously undergone palliative cardiac surgery
- Patients with impaired renal function tests
- Patients who were unsuitable for surgical repair
- Patients with critical comorbidities

All patients were evaluated with transthoracic echocardiography (TTE) during the diagnostic and follow-up process. In addition to echocardiography, other imaging modalities such as CT angiography (CTA) and conventional catheter angiography (CCA) were used to provide a detailed anatomical assessment before surgery.

Our study was a single-center, retrospective study. We included patients under 1 year of age who underwent complete repair between January 2021 and December 2024. Patients over one year of age, TOF patients with pulmonary atresia, TOF patients with atrioventricular septal defect, TOF patients with hypoplastic ventricles, patients who had previously undergone palliative cardiac surgery, patients with impaired renal function tests, patients who were unsuitable for surgical repair, and patients with critical comorbidities were excluded from the study. All patients were evaluated with transthoracic echocardiography (TTE) during the diagnostic and follow-up process. In addition to echocardiography, other imaging modalities such as CT angiography (CTA) and conventional catheter angiography (CCA) were used to provide a detailed anatomical assessment before surgery.

We compared the results of CTA and CCA performed in addition to TTE to evaluate the accuracy of anatomy before total correction in patients with TOF. Demographic variables, including age, sex, height, weight, and body surface area (BSA = 0.007184 × height (cm)^0.725^ × weight (kg)^0.425^; calculated using the Du Bois method) were documented.

Echocardiographic evaluation was performed using the Philips Affiniti 50 cardiac ultrasound system (Philips Affiniti 50 Cardiac Ultrasound, Bothell, WA, USA) with a 9 and 12 MHz probe. All patients underwent echocardiography using standardized protocols according to the guidelines of the American Society of Echocardiography. Standard views of the pediatric echocardiogram were recorded, including parasternal (long and short axis), apical (four and five chambers), subcostal, and suprasternal views. Cardiac morphology was evaluated in the direction of blood flow using a segmental approach. Atrial situs, venoatrial connection (systemic and pulmonary venous return), atrium-ventricular (AV) connections, ventricles, ventricular-great artery (VA) connection, the spatial position of great arteries, intracardiac defects, and extracardiac vascular anomalies were examined as the main components of this approach. The pattern of coronary artery anatomy in patients with TOF was carefully identified in the parasternal short-axis views as described in the American Society of Echocardiography report. In the ideal imaging position, both semilunar valves, as seen in cross-sectional views, and both coronary artery origins were visualized. Two-dimensional transthoracic echocardiography (Philips Epix 3D device, Amsterdam, Netherlands) was performed by two pediatric cardiologists with more than 10 years of experience in the evaluation of congenital heart disease. During the two-dimensional transthoracic echocardiographic evaluation of the infants, no additional sedative medication was administered.

Cardiac CT was performed on a single-source 64-slice CT (aquillion ONE, GENESIS Edition Canon medical systems, Tochigi, Otowara, Japan ) using a wide detector aperture (16 cm), an iterative reconstruction algorithm, Adaptive Iterative Dose Reduction (AIDR 3D Enhanced). A prospective ECG-gated technique was performed during one cardiac cycle in all patients. All images were acquired in axial mode (rotation time: 0.275 s, scan range: 80–120 mm). The tube current was set by automatic exposure control. A low kV value (80 kV) was selected to maximise the iodine contrast-to-noise ratio for all children. All patients were injected intravenously with an iodinated contrast agent (Kopaq 300 mg I/mL, Onko & Kocsel Pharmaceuticals, Kocaeli, Turkey) at a rate of 1.5 mL per kg, followed by 10–20 mL of saline solution with a dual-head power injector (Bayer HealthCare, MEDRAD Europe, Beek, Netherlands). The injection rate was 0.7–0.9 mL/kg, depending on the catheter and patient size. The contrast agent was used undiluted for the procedure. All cardiac CT images were obtained without breath-hold and without sedation for the study. All examinations were performed under the direction of an attending cardiac radiologist at our hospital. All images were acquired during the first pass of contrast through the anatomical structures of interest. Targets for the center of the acquisition window were set at 45% of the R–R interval in patients (all patients with heart rates higher than 90 beats/min). During other phases of low interest, the operation is stopped. The best motion-free cardiac phase adjacent to the predefined cardiac phase was individually selected from the raw data for each patient by the attending radiologist. Axial images for all cardiac CT images were transferred to a post-processing workstation. Image reconstruction was performed with a slice thickness of 0.5 mm and the standard reconstruction kernel. Iterative reconstruction was performed with AIDR. Reconstructed images were acquired using multiplanar reconstruction (MPR), maximum intensity projection (MIP), and 3D volume rendering (VR) techniques.

Cardiac CT and associated findings were compared with ECHO and surgical findings. Radiation exposure parameters, including imaging area, dose-length product, and volumetric CT dose index, were recorded for each patient who underwent cardiac CT. The CT dose index, dose-length product, and conversion factor were expressed for a 32 cm body phantom reference. The ED was calculated from the DLP multiplied by 2 to apply to the 16 cm phantom. The cardiac CT effective dose was calculated from the dose-length product multiplied by the conversion factor. The specific dose-length product conversion coefficients for neonates and infants were 0.039 mSv/(mGy.cm) DI for each age group. CTA was performed by a pediatric cardiovascular radiologist with at least 15 years of experience in congenital heart imaging.

Cardiac catheterization protocol for pulmonary vascular measurements Cardiac catheterization was performed via femoral access under anesthesia on Philips^®^ Artis biplane AZURION 7 B12 (Philips Medical Systems, Eindhoven, Netherlands). Intravenous midazolam and/or fentanyl were given by a cardiac anesthetist when sedation was required. The patients were adequately hydrated before and after the procedure, and renal function tests were carried out before and after each procedure. Pressure and oxygen saturations in different cardiac chambers, the aorta, and the vena cava were obtained using fluid-filled catheters. Isoosmolar non-ionic contrast (iodixanol) media was used for angiocardiography at a maximum dose of 4 mL/kg. Right ventricular angiograms in the anteroposterior view with cranial angulation and in the left anterior oblique view were obtained to delineate the RV outflow tract, pulmonary artery anatomy, and ventricular contractility. Left ventricular angiogram in the left anterior oblique view with cranial angulation was obtained to evaluate ventricular size and function, location of the VSD, presence of any additional VSD, and degree of aortic override. Aortic root angiography was undertaken to delineate the arch laterality and major aortopulmonary collaterals and assess descending thoracic aorta diameter at the level of the diaphragm. Levo-phase was used to delineate pulmonary venous drainage. Selective right coronary angiography was used to confirm its origin and any branch crossing over the RVOT. Measurements of radiation dose are reported for each case by the catheterization system of Philips ^®^ Artis biplane AZURION 7 B12 (Philips Medical Systems, Eindhoven, Netherlands) and categorized as those that are obtained through fluoroscopy alone versus those obtained through digital acquisition. Conventional catheter angiography (CCA) was performed by a pediatric cardiologist with more than 10 years of experience in the evaluation of congenital heart disease.

To monitor radiation exposure, the patient dose was indirectly recorded using standard techniques, including total fluoroscopy time (minutes), air kerma (mGy), and dose-area product (DAP; µGy m^2^). Air kerma dose is the dose measured in air at a fixed distance from the X-ray tube and is the best surrogate of the radiation absorbed at the skin surface at the site of the beam entrance. DAP; µGy m^2^ is the instantaneous air kerma dose times the X-ray field area, reflecting the total dose given to the patient. Cardiac catheterization with angiography data were independently analyzed by a cardiologist blinded to CTA and ECHO findings.

The three imaging modalities were used to evaluate the parameters of Right ventricular (RV) to pulmonary artery (PA) continuity; right ventricular outflow tract (RVOT) anatomy, right ventricular (RV) size, and location of RVOT obstruction; pulmonary annulus, branching patterns, and dimensions of the main pulmonary artery (MPA), right pulmonary artery (RPA), and left pulmonary artery (LPA); the size and position of the left ventricle (LV), the size of the ventricular septal defect (VSD), and the presence of additional VSDs; the anatomy of the coronary arteries, including their origins and any major coronary artery or branch that crosses the RVOT; the anatomy and branching patterns of the aortic arch; and the presence of major aortopulmonary collateral arteries (MAPCAs) arising from any part of the aorta or its branches. The Nakata index was calculated as the sum of the cross-sectional areas of the right pulmonary artery (RPA) and left pulmonary artery (LPA) divided by the body surface area (BSA). The McGoon ratio is determined by measuring the diameters of the right pulmonary artery (RPA) and the left pulmonary artery (LPA), summing these values, and dividing the total by the diameter of the descending aorta (DAO), which is measured at the level of the diaphragm.

The dimensions of the right pulmonary artery (RPA) and left pulmonary artery (LPA) were measured immediately proximal to the origin of the upper lobe branches, while the diameter of the descending aorta (DA) was measured at the level of the diaphragm. The Nakata index, the McGoon ratio, and age-adjusted Z scores for pulmonary annulus and pulmonary arteries were used as baseline measures in this study. All measured parameters were indexed to BSA. Z-scores normalized for age were calculated for the pulmonary annulus, RPA, and LPA using the Detroit reference data. Data of patients evaluated by transthoracic echocardiography (TTE), conventional catheter angiography, and cardiac CT angiography were compared. We compared our imaging findings with the surgical outcomes.

To ensure consistency in image interpretation across different modalities, all imaging assessments were conducted independently. Radiologists analyzing CTA and cardiologists evaluating CCA were blinded to the results of the other imaging modalities to prevent bias. Each imaging study was reviewed separately by specialists with extensive experience in congenital heart disease imaging, following standardized protocols.

Continuous variables are presented as mean standard deviation. Categorical variables are presented as percentages. Statical analyses were performed to compare pulmonary annulus and other pulmonary artery measurements (diameter and Z-score) obtained through different methods (ECHO, CTA, and CCA). Diagnostic performance metrics, including sensitivity, specificity, positive predictive value (PPV), and negative predictive value (NPV), were calculated for each imaging modality using standard formulas. To ensure that the selection of statistical tests was appropriate for our data, we first assessed the normality of the distributions using the Shapiro–Wilk test. If the data followed a normal distribution (*p* > 0.05), one-way ANOVA was used for multiple group comparisons, while paired t-tests were used for pairwise analyses. If the normality assumption was not met (*p* < 0.05), the Kruskal–Wallis’s test was used for group comparisons and the Mann–Whitney U test for pairwise comparisons. After ANOVA, Tukey’s HSD test was used for pairwise comparisons. AUC values provide a more robust interpretation of the diagnostic performance of the imaging modalities used in our study. All statistical analyses were performed using SPSS version 25.0 (SPSS Inc., Chicago, IL, USA), and a *p*-value of <0.05 was considered statistically significant.

## 3. Result

The baseline clinical characteristics of the 127 TOF patients who underwent complete repair surgery are summarized in Table 1. After preoperative transthoracic echocardiography, 68% of patients (n = 86) underwent detailed anatomic evaluation with CTA and 32% (n = 41) with CCA. There were no missing data in this study.

Among the patients, 62% were male and 38% were female. The mean age of the patients was 5.81 ± 2.15 months, the mean oxygen saturation was 88.07 ± 7.89%, and the mean weight was 7.44 ± 5.90 kg. The diagnostic performance of echocardiography (ECHO), conventional coronary angiography (CCA), and computed tomography angiography (CTA) were evaluated by comparing their sensitivity (SNS), specificity (SPS), positive predictive value (PPV), negative predictive value (NPV), and area under the ROC curve (AUC) to surgical diagnosis, which served as the gold standard, with the results shown in Table 2. CCA showed perfect sensitivity and specificity for detecting coronary anomaly and MAPCA, making it the most reliable imaging modality. CTA also performed highly, with a high AUC, whereas ECHO had a lower PPV, especially for MAPCA, suggesting its limited value in confirming true positive cases.

On TTE, the main pulmonary artery diameter was 9.16 ± 2.10 mm with a z-score of 1.46 ± 0.82, the right pulmonary artery diameter was 7.39 ± 1.57 mm with a z-score of 0.59 ± 1.60, and the left pulmonary artery diameter was 7.58 ± 1.85 mm with a z-score of 0.93 ± 1.12. In patients with preoperative CCA, the pulmonary annulus measured 8.38 ± 1.32 mm with a z-score of −1.29 ± 1.68. The main pulmonary artery measured 9.24 ± 2.05 mm with a z-score of 1.49 ± 0.76. The right pulmonary artery measured 7.41 ± 1.47 mm with a z-score of 0.60 ± 1.51, and the left pulmonary artery measured 7.73 ± 1.58 mm with a z-score of 1.09 ± 0.98. The Nakata index was 318.25 ± 139.25, and the McGoon ratio was 1.85 ± 0.19. The procedure duration for CCA was 20.73 ± 11.12 min. The contrast volume was 40.17 ± 13.26 mL, and the radiation dose was 5.48 ± 1.62 mSv. Among patients with CCA, 16% (n = 5) had small aortopulmonary collateral arteries (APCAs) measuring <2 mm, and 9% (n = 3) had large aortopulmonary collateral arteries (MAPCAs) measuring >2 mm. Coronary anomalies were identified in 13% (n = 4) of patients, with the most common anomaly being a conal branch crossing the RVOT.

**Figure 1:**
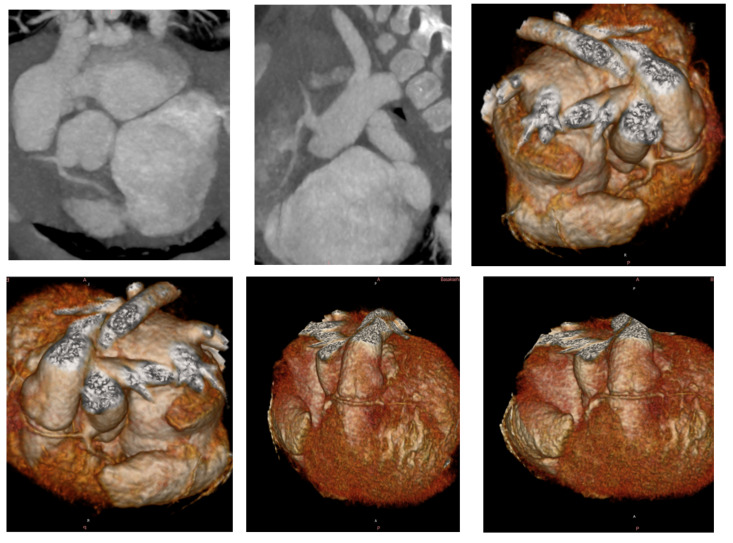
A patient with a single coronary origin and crossing the right ventricular outflow tract (RVOT).

**Figure 2:**
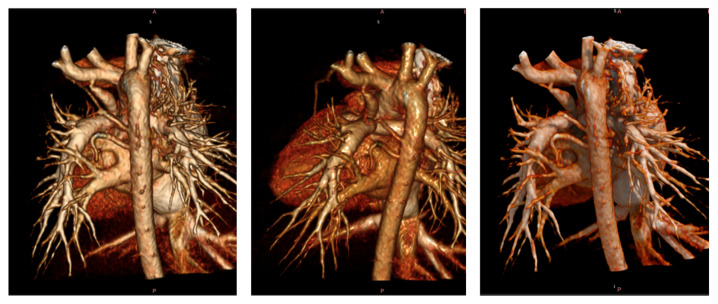
A patient with a MAPCA (major aortopulmonary collateral artery) originating from the descending aorta.

In patients evaluated with CTA, the pulmonary annulus measured 8.44 ± 1.75 mm with a Z-score of −1.25 ± 1.69. The main pulmonary artery measured 9.22 ± 2.04 mm with a Z-score of 1.47 ± 0.74. The right pulmonary artery measured 7.43 ± 1.41 mm with a Z-score of 0.63 ± 1.43, and the left pulmonary artery measured 7.79 ± 1.61 mm with a Z-score of 1.12 ± 1.02. The McGoon ratio was 1.85 ± 0.19, and the Nakata index was 318.25 ± 139.25. The duration of the procedure was 3.1 ± 0.58 min. The contrast volume was 7.70 ± 2.38 mL, and the radiation dose was 1.19 ± 0.22 mSv. The comparative analysis of different imaging modalities used in the preoperative evaluation of Tetralogy of Fallot is shown in Table 3. In patients with CTA, coronary anomalies were identified in 10% (n = 9) of patients, with the most common anomaly being a conal branch crossing the RVOT. A patient with a single coronary origin and an anomalous course crossing the RVOT is shown in Figure 1. Among patients with CTA, 14% (n = 12) had small aortopulmonary collateral arteries (APCAs) measuring <2 mm, and 10% (n = 9) had large aortopulmonary collateral arteries (MAPCAs) measuring >2 mm. A patient with a large aortopulmonary collateral artery (MAPCA) originating from the descending aorta is shown in Figure 2.

Both CCA and CTA were successful in the evaluation of the degree of aortic override and perimembranous VSDs. However, CTA failed to detect additional muscular VSDs in three patients. The right ventricle (RV) was evaluated using both techniques. The location of hypertrophic muscle bundles and the level of RVOT obstruction (infundibular, subinfundibular, valvular, supravalvular, or multilevel) were evaluated similarly by both techniques.

There was no statistically significant difference in the measurements of pulmonary annulus diameter and z-score, main pulmonary artery diameter and z-score, and right pulmonary artery diameter and z-score across the imaging methods. However, there was a statistically significant difference in the measurements of left pulmonary artery (LPA) diameter and z-scores between the imaging methods, with echocardiography (ECHO) measuring a smaller LPA diameter compared to other imaging techniques (*p* < 0.05). Additionally, there were statistically significant differences in procedure duration, contrast volume, and radiation dose (*p* < 0.05). Statistically significant differences in the Nakata index and the McGoon ratio were not found. With both imaging modalities, coronary artery abnormalities were detected and well- characterized at similar frequencies.

## 4. Discussion

Transthoracic echocardiography (TTE) is the primary imaging modality for the preoperative evaluation of patients with Tetralogy of Fallot (TOF). Although it provides an excellent assessment of intracardiac morphology, it is limited in its ability to evaluate extracardiac morphology, such as the anatomy of the pulmonary artery and its branches, the course of the coronary arteries, and the major aortopulmonary collateral arteries (MAPCAs). Conventional catheter angiography has been the traditional method of evaluating these structures. The most common reason for preoperative evaluation with CCA in TOF patients is to define the pulmonary artery and its anatomy. However, with recent technological advances, cardiac computed tomography (CT) angiography has become the preferred modality due to its high-quality imaging, reduced radiation dose, and lower contrast requirements.

Although conventional angiography has traditionally been used for the preoperative evaluation of TOF patients, it has not been the gold standard due to its invasive nature, potential access point complications, reliance on operator skill, and exposure to large amounts of contrast and significant radiation. The relatively non-invasive nature of CTA, combined with its ability to use significantly less radiation and contrast, allows for multiple manipulable images to be obtained with a single contrast injection. In addition, the advanced technological infrastructure and workstation capabilities allow for the generation of multiple projections, making CTA a reliable, non-invasive alternative to CCA. Naveen Garg and colleagues concluded in their study that multidetector computed tomographic angiography (MDCT) is a reliable, non-invasive alternative to cardiac catheterization for the preoperative evaluation of Tetralogy of Fallot. In conclusion, we found CTA to be an excellent imaging modality for defining RVOT and PA anatomy in the preoperative evaluation of TOF patients.

In a study by Xi-Ming Wang and colleagues, 64-slice spiral CT showed better results than transthoracic echocardiography in the preoperative evaluation of TOF patients (%95 vs. %82). However, this study was performed in a small cohort of patients (n = 12) [14]. In our study, we had similar results with a significantly larger patient population. Hayabuchi et al. and Kasar et al. reported a complete correlation between computed tomography angiography (CTA) and conventional catheter angiography (CCA) in measuring the diameters of the main pulmonary artery and its branches [15,16]. Similarly, in our study, the diameters and z-scores of the main pulmonary artery and its branches were comparable between the two imaging methods. In our study, the diameters and z-scores of the main pulmonary artery and its branches were also found to be statistically similar between the two imaging modalities. In our study, the measurements of the pulmonary annulus, pulmonary arteries, and their branches obtained by CTA showed a very strong correlation with the CCA data.

Several indices, such as the Nakata index and the McGoon ratio, have been used to predict preoperative surgical outcomes in TOF patients. These indices (Z-scores, the Nakata index) were statistically similar between CCA and CTA in our study. A study by Meinel et al. reported results that were consistent with ours. In cases where further evaluation or vascular intervention is required, cardiac catheterization is appropriate. Pre-evaluation with CTA reduces the number of angiographic projections needed, resulting in less radiation exposure, less contrast media use, and shorter procedure times.

The McGoon ratio, which standardizes the comparison between pulmonary artery dimensions and systemic vascular capacity, is a reliable measure for assessing the pulmonary vascular bed. Its simplicity and clinical importance have made the McGoon ratio a fundamental parameter in the management of TOF and other congenital heart defects associated with pulmonary artery hypoplasia. This measurement is critical for preoperative planning, helping surgeons determine if pulmonary arteries are developed enough for effective circulation after repair. In our study, the McGoon ratıo was consistent with the literature and statistically similar with all three imaging modalities. The Nakata Index is an important measure of pulmonary artery size relative to body surface area (BSA) in patients with congenital heart defects such as Tetralogy of Fallot (TOF). It is calculated by summing the cross-sectional areas of the right and left pulmonary arteries (RPA and LPA) and dividing the sum by the patient’s BSA. This index is critical to surgical planning as it helps predict postoperative outcomes and guides decisions regarding the feasibility of complete repair or the need for staged procedures in cases of pulmonary artery hypoplasia. The results of the Nakata index with both ımagıng modalıtıes were similar and did not differ statistically.

In our study, measurements of the left pulmonary artery (LPA) were statistically different between echocardiography (ECHO) and both cardiac catheterization (CCA) and computed tomography angiography (CTA). The LPA is naturally more difficult to visualize than the right pulmonary artery (RPA). This mismatch is primarily due to the abrupt angulation of the LPA along its long axis and its posterior orientation, which complicates its visualization. Anatomically, the RPA generally follows a consistent course anterior to the right main bronchus, making it well visualized on cross-sectional imaging at the level of the tracheal bifurcation, especially with CTA. In contrast, the LPA is often difficult to locate due to its inconsistent position and variable orientation. Given these challenges, visualization of the LPA is more prone to interpretation failure when evaluated by CTA than it is with the RPA. To address this limitation, scan modifications such as gantry angulation adjustments or sagittal reconstruction may be beneficial to improve visualization. The study by Amarendu Kumar and colleagues compared measurements obtained by transthoracic echocardiography (ECHO), computed tomography angiography (CTA), and conventional cardiac angiography (CCA) in patients with Tetralogy of Fallot physiology over 10 kg before surgical correction [8]. The study showed that CTA and CCA were more effective than ECHO; however, ECHO was shown to have difficulty in accurately assessing the left pulmonary artery (LPA) compared to the other modalities. Similarly, in our study, the LPA and its branches were measured to be smaller on ECHO compared to CTA and CCA, likely due to the difficulty in visualizing the LPA on ECHO. In addition, the study by Kasar et al. showed that echocardiography underestimated left pulmonary artery (LPA) diameter in unrepaired TOF patients compared with computed tomography angiography, thus highlighting its potential to lead to preoperative evaluation and surgical decision-making inaccuracies [15].

In our study, postoperative surgical outcomes identified coronary anomalies in a certain percentage of cases, with both CCA and CTA demonstrating high accuracy in identifying coronary anatomy. However, a thin conal branch could not be identified in one patient by CCA and in two patients by CTA. The surgical technique was not altered in any of the patients. Although CTA and CCA showed similar rates of identification of coronary anatomy, selective coronary angiography may be necessary for CCA to identify coronary anatomy, and it has a higher risk compared to CTA. In a study by Vastel-Amzallag and colleagues, CTA showed 100% sensitivity and 100% specificity in the identification of coronary artery abnormalities in TOF patients [17]. Similarly, Kasar and colleagues reported that CTA had an accuracy rate of 96% when compared with surgical results [15]. In our study, coronary artery anomaly was detected at a similar rate. In a study conducted by Sangita Kapur and colleagues, coronary anomalies were reported to have an incidence ranging from 4% to 36% [18]. In a study by Zsófia Kakucs and colleagues, the overall prevalence of coronary artery anomalies (CAAs) was 7.61% (8 out of 105 cases), with the anomalous origin and course of coronary arteries across the RVOT (prepulmonic course) identified in 5.71% of cases (six patients), representing 75% of CAAs; similarly, in our study, CAAs were detected in 9% of cases, with the most common anomaly being the across-the-RVOT coronary anomaly [19].

Preoperative evaluation of MAPCAs is critical in patients with Tetralogy of Fallot as it provides detailed anatomical and functional information to ensure optimal pulmonary blood flow during corrective surgery. Accurate assessment minimizes surgical risks and improves long-term outcomes by identifying collateral vessel anatomy and potential complications. In the study by Renata Junqueira Moll Bernardes and colleagues, MR angiography was shown to provide better visualization and characterization of MAPCAs compared to conventional angiography. Venkateswara Rao and colleagues also demonstrated that gadolinium-enhanced three-dimensional magnetic resonance angiography may provide a non-invasive alternative to diagnostic catheterization for the evaluation of pulmonary artery anatomy in patients with Tetralogy of Fallot [10,20].

Identification of MAPCAs in the preoperative period is crucial because of their association with prolonged ICU stay, prolonged hospital stay, postoperative bleeding, and heart failure. Depending on their size and location, MAPCAs can be occluded during surgery or preoperatively with coils during CCA. The ability of CTA to visualize MAPCAs in a single imaging session is a significant advantage, whereas CCA may require multiple injections. In our study, the detection rate of MAPCAs was similar between CCA and CTA, which is in agreement with the literature. Patients who underwent additional procedures, such as MAPCA coil embolization, after imaging with conventional angiography were excluded from the study due to the increased procedure time and radiation dose.

In the study by E. N. Yakoumakis et al., diagnostic procedures during CCA were performed with an average radiation dose of 5 mSv [21]. Similarly, A. Karambatsakidou et al. reported an average dose of 8 mSv in 1-year-old children. Another study found that CTA exposes children to significantly lower radiation (0.76 mSv) compared to CCA (13.4 mSv) [22]. In our study, comparable radiation doses were observed, aligning with these findings. Felix G. Meinel, MD, and colleagues demonstrated that CT angiography (CTA) is highly accurate for evaluating Tetralogy of Fallot with pulmonary atresia (ToF-PA) and MAPCAs, with strong correlation to catheterization findings and significantly lower radiation dose (0.9 mSv vs. 14.4 mSv) [20]. In our study, radiation dose, contrast dose, and procedure time were statistically significantly lower with CTA compared to CCA. Similarly, in our study, the radiation dose was significantly lower in CTA compared to CCA. MDCT was found to provide an equivalent assessment of intracardiac and pulmonary anatomy while offering better delineation of coronary anatomy, with fewer complications and no need for hospitalization.

In four patients in our study, additional VSDs were not detected by CTA. CTA may have encountered difficulties in detecting small muscular VSDs in patients due to several factors. Compared with echocardiography, its limited spatial resolution may make it more difficult to identify very small defects within the trabeculated muscular septum. Motion artifacts resulting from rapid cardiac motion, especially in pediatric patients, can reduce image clarity and obscure fine structural details. Inadequate contrast opacification during certain cardiac phases can also contribute to reduced visibility of small defects. In addition, partial volume averaging in thin-slice CT imaging may obscure or underestimate the size of small VSDs. In addition, the lower volume of contrast used in CTA, along with images acquired predominantly in the systolic phase due to ECG gating, may further limit the detection of small intracardiac shunts. While CT is generally an excellent modality for anatomical assessment, its performance in the evaluation of functional parameters and intracardiac flow is less than that of echocardiography.

In addition, CTA allows evaluation of the lung parenchyma and additional pulmonary pathologies. In our study, 12% of patients had abnormal lung parenchyma, with subsegmental atelectasis being the most common finding. In our study, two patients experienced access site hematoma with CCA, increasing the length of hospital stay for these patients. However, if an additional preoperative procedure, such as MAPCA coil embolization, is required after CTA, CCA is preferred.

While our study focused primarily on the diagnostic efficacy and safety profiles of computed tomography angiography (CTA) and conventional catheter angiography (CCA), we recognize that cost, accessibility, and healthcare infrastructure play an important role in their clinical use. CTA is generally more accessible and cost-effective in high-resource settings due to its non-invasive nature, shorter procedure time, and reduced need for sedation. However, in low-resource settings, limited access to advanced CT scanners and post-processing software may favor the continued use of CCA despite its invasiveness. In addition, both modalities require specialized personnel for image acquisition and interpretation, which may further influence feasibility in different healthcare systems. In addition, as the use of iodinated contrast in CTA has similar risks to CCA, the choice between CTA and CCA should be carefully considered in patients with renal impairment or contrast allergy.

## 5. Limitations

This study has several limitations that should be acknowledged. The single-center, retrospective design may affect the applicability of the results to a larger patient population. Only one imaging modality—either CT angiography (CTA) or conventional catheter angiography (CCA)—was used for preoperative evaluation per patient, rather than both modalities being used for direct comparison. Although the study included a significant number of patients, the sample size remains relatively small, and a larger multicenter study would provide more robust and generalizable conclusions. The study focused exclusively on patients with simple Tetralogy of Fallot, excluding those with more complex anatomical variations, which may affect the applicability of the findings to a larger TOF population. In addition, long-term surgical and clinical outcomes were not evaluated, as the study focused primarily on preoperative imaging and short-term postoperative outcomes.

Future research with a prospective, multicenter approach, larger sample sizes, and long-term follow-up would be beneficial to further validate these findings and assess their broader clinical implications.

## 6. Conclusions

Transthoracic echocardiography (TTE) serves as the primary imaging modality for the initial evaluation of patients with Tetralogy of Fallot (TOF). Echocardiography and computed tomography angiography (CTA) are considered complementary modalities in the preoperative assessment of TOF patients. While TTE is optimal for intracardiac anatomy and functional analysis, CTA excels in the assessment of extracardiac structures. In the preoperative evaluation of TOF patients, we advocate the use of CTA in combination with TTE, particularly for extracardiac imaging, owing to its significant benefits. These benefits include low-dose radiation (80 kV), shorter procedure durations, reduced overall radiation exposure, and fewer access-related complications. Additionally, electrocardiogram (ECG)-triggered imaging enhances synchronization with cardiac motion, mitigating motion artifacts. Volume computed tomography allows for comprehensive imaging of extensive anatomical regions within a single scan and can be performed without anesthesia, thereby enhancing patient safety and comfort.

Moreover, for patients requiring interventional procedures, such as major aortopulmonary collateral arteries (MAPCA) coiling, the combined use of CTA and conventional catheter angiography (CCA) is recommended to improve both pre-procedural planning and procedural outcomes. While CTA offers detailed anatomical visualization, CCA remains crucial for dynamic imaging and therapeutic intervention.

## Figures and Tables

**Table 1:** The baseline clinical characteristics.

Sex (male)	%62 (n = 78)
Age (month)	5.81 ± 2.15
Weight (kg)	7.44 ± 5.90
Weight (percentile)	34.83 ± 15.90
Weight (SDS)	−0.15 ± 1.12
Height (cm)	120.81 ± 14.15
Height (percentile)	40.42 ± 21.23
Height (SDS)	0.32 ± 1.38
BMI kg/m²	18.4 ± 4.35
BMI (percentile)	22.45 ± 11.89
BMI (SDS)	−0.67 ± 1.38
BSA(kg/m^2^)	0.38 ± 0.13
Saturation (spo2)	88.07 ± 7.89
Access site complication (CCA)	%10 (n = 4)
Cyanotic spell during procedure (CCA)	%5 (n = 2)

**Table 2:** Compare diagnostic performance of imaging modalities.

		Diagnosis	Surgical diagnosis						
			(+)	(−)	SNS	SPS	PPV	NPV	AUC
Coronary anomaly	ECHO	(+)	9	13	60%	88%	40%	95%	85%
		(−)	6	99					
	CCA	(+)	4	0	100%	100%	100%	100%	100%
		(−)	0	37					
	CTA	(+)	8	1	80%	98%	89%	95%	96%
		(−)	2	75					
APCA (<2 mm)	ECHO	(+)	17	8	78%	92%	69%	93%	89%
		(−)	5	96					
	CCA	(+)	8	1	89%	97%	89%	97%	95%
		(−)	1	31					
	CTA	(+)	10	2	77%	97%	83%	97%	94%
		(−)	3	71					
MAPCA (>2 mm)	ECHO	(+)	9	16	75%	86%	37%	97%	84%
		(−)	3	98					
	CCA	(+)	3	0	100%	100%	100%	100%	100%
		(−)	0	38					
	CTA	(+)	8	1	89%	99%	89%	99%	97%
		(−)	1	76					

SNS: Sensitivity, SPS: Specificity, PPV: Positive Predictive Value, NPV: Negative Predictive Value, AUC: Area Under the ROC Curve, ECHO: Echocardiography, CCA: Conventional Coronary Angiography, CTA: Computed Tomography Angiography, (+): Identified, (−): Not Identified.

**Table 3:** Comparison of Imaging Modalities in Tetralogy of Fallot Assessment.

Anatomy	ECHO (n = 127)	CTA (n = 86)	CCA (n = 41)	*p*-Value
**Pulmonary annulus**				
Diameter (mm)	8.32 ± 1.72	8.44 ± 1.75	8.38 ± 1.32	ECHO vs. CTA = 0.02 ECHO vs. CCA = 0.57 CCA vs. CTA = 0.057
Z-score	−1.31 ± 1.67	−1.25 ± 1.69	−1.29 ± 1.68	ECHO vs. CTA = 0.69 ECHO vs. CCA = 0.91 CCA vs. CTA = 0.76
**Main pulmonary artery (MPA)**				
Diameter (mm)	9.16 ± 2.10	9.22 ± 2.04	9.24 ± 2.05	ECHO vs. CTA = 0.17 ECHO vs. CCA = 0.72 CCA vs. CTA = 0.32
Z-score	1.46 ± 0.82	1.47 ± 0.74	1.49 ± 0.76	ECHO vs. CTA = 0.14 ECHO vs. CCA = 0.87 CCA vs. CTA = 0.16
**Right pulmonary artery (RCA)**				
Diameter (mm)	7.39 ± 1.57	7.43 ± 1.41	7.41 ± 1.47	ECHO vs. CTA = 0.91 ECHO vs. CCA = 0.82 CCA vs. CTA = 0.81
Z-score	0.59 ± 1.60	0.63 ± 1.43	0.60 ± 1.51	ECHO vs. CTA =0.59 ECHO vs. CCA = 0.78 CCA vs. CTA = 0.39
**Left pulmonary artery (LPA)**				
Diameter (mm)	7.58 ± 1.85	7.79 ± 1.61	7.73 ± 1.58	ECHO vs. CTA = 0.009 ECHO vs. CCA = 0.043 CCA vs. CTA = 0.51
Z-score	0.93 ± 1.12	1.12 ± 1.02	1.09 ± 0.98	ECHO vs. CTA =0.0015 ECHO vs. CCA = 0.005 CCA vs. CTA = 0.67
Nakata index		310.69± 142.2	318.2 ± 139.2	CCA vs. CTA = 0.27
McGoon ratio		1.83 ± 0.21	1.85 ± 0.19	CCA vs. CTA = 0.69
Procedure Duration		3.1 ± 0.58	20.73 ± 11.12	CCA vs. CTA < 0.01
Contrast Volume		7.70 ± 2.38	40.17 ± 13.26	CCA vs. CTA < 0.01
Radiation dose (mSv)		1.19 ± 0.22	5.48 ± 1.62	CCA vs. CTA < 0.01

## Data Availability

The data that support the findings of this study are available from the corresponding author upon reasonable request.
